# Pyrroloquinoline Quinone Improves Cognitive-Related Behavioral Performance Associated with Enhanced Mitochondrial Bioenergetics in Naturally Aged Mice

**DOI:** 10.3390/antiox15080921

**Published:** 2026-07-24

**Authors:** Yun Yan, Di Deng, Chunxia Tan, Qi Lu, Jiutang Sun, Shaoliang Wu, Zhanhua Jiang, Yibo Li, Tao Lu

**Affiliations:** 1 School of Life Sciences, Beijing University of Chinese Medicine, Beijing 102488, China; 20140422015@bucm.edu.cn (Y.Y.); 20250941121@bucm.edu.cn (D.D.); 20230941093@bucm.edu.cn (C.T.); 20241221014@bucm.edu.cn (Q.L.); 20221221019@bucm.edu.cn (J.S.); 20241221007@bucm.edu.cn (S.W.); 20250321144@bucm.edu.cn (Z.J.); 2 Shenzhen Maternity and Child Healthcare Hospital, Women and Children’s Medical Center, Southern Medical University, Shenzhen 518000, China

**Keywords:** pyrroloquinoline quinone, natural aging, cognitive decline, mitochondria function, oxidative stress

## Abstract

We investigated whether pyrroloquinoline quinone (PQQ) could improve cognitive performance in twenty-month-old naturally aged mice and explored the potential mechanisms involved. Results showed that PQQ supplementation improved spatial working memory and recognition memory without inducing anxiety-like behavior, and was associated with better preservation of hippocampal neuronal integrity. In HT-22 hippocampal neuronal cells, PQQ reduced reactive oxygen species (ROS) accumulation, restored mitochondrial membrane potential, and enhanced mitochondrial respiratory capacity. Hippocampal transcriptomic analysis and upstream regulator prediction identified sirtuin 1 (SIRT1) as a major regulator associated with the PQQ-induced transcriptional response, while uncoupling protein 2 (UCP2) emerged as a candidate downstream mitochondrial effector. Consistently, PQQ increased hippocampal SIRT1 protein expression and downregulated UCP2 at both mRNA and protein levels. Pharmacological inhibition of SIRT1 attenuated the PQQ-induced increase in ATP production and partially weakened the regulatory effect of PQQ on UCP2, supporting the involvement of SIRT1 in PQQ-associated mitochondrial bioenergetic regulation. Collectively, these findings indicate that PQQ improves cognitive-related behavioral performance in naturally aged mice and is associated with mitochondrial bioenergetic regulation, and suggest that modulation of a SIRT1–UCP2-associated pathway may contribute to its neuroprotective effects.

## 1. Introduction

It is widely recognized that with increased age, cognitive impairment becomes a common and significant complication, with gradual declines typically described in learning capacity, memory performance, and executive function [1]. Of the cognition-related brain regions, the hippocampus has been found to be particularly vulnerable to age-associated damage, and this vulnerability is believed to result from its exceptionally high metabolic demand and strong reliance on mitochondrial energy production [2]. Aging is also accompanied by broad transcriptomic remodeling and dysregulation of metabolic homeostasis. A recent cross-species transcriptome meta-analysis further identified aberrant RNA processing as a common feature of aging, particularly in the brain [3].

Mitochondria play a critical role in neuronal function, supporting ATP production, maintaining redox balance, and regulating intracellular calcium signaling [4]. With aging, however, mitochondrial homeostasis progressively deteriorates, leading to an impairment in energy metabolism and increased oxidative stress [5]. These changes impede the ability of neurons to satisfy their high metabolic demands, thereby interfering with synaptic plasticity, compromising neuronal structural integrity, and contributing to cognitive decline [6]. There is growing evidence that mitochondrial dysfunction is closely linked with hippocampal degeneration and cognitive decline during aging [7,8]. Thus, strategies aimed at preserving mitochondrial function and metabolic balance have emerged as an important focus in efforts to mitigate cognitive decline associated with aging [9].

Pyrroloquinoline quinone has been identified as a cofactor in some bacterial metabolic processes [10,11]. Recent studies have established the antioxidant properties of the compound and its influence on mitochondrial functions in a number of different cell types [12,13,14]. There have also been a number of studies indicating the compound can have a stimulating effect on mitochondrial biogenesis while providing a degree of protection from the effects of oxidative damage in a number of different tissues [15,16]. Within the nervous system, PQQ has been reported to exert neuroprotective effects in experimental models characterized by oxidative stress or neurodegenerative pathology [17]. Nevertheless, whether and how PQQ modulates mitochondrial function to alleviate cognitive decline during the process of natural aging remains insufficiently defined.

Sirtuin 1 (SIRT1) is a member of the sirtuin family of NAD^+^-dependent deacetylases [18]. SIRT1 acts as an essential regulator for cellular energy homeostasis, mitochondrial health, and stress resistance [19]. During aging, both the expression level and enzymatic activity of SIRT1 tend to decline [20], a change that has been linked to mitochondrial dysfunction, elevated oxidative stress, and disruption of metabolic homeostasis [21]. However, in animal models of aging and neurodegenerative diseases, SIRT1 activity enhancement improves neuronal function and cognitive performance [22].

Uncoupling protein 2 (UCP2) is an inner mitochondrial membrane protein involved in the regulation of mitochondrial proton conductance. This protein partially dissipates the electrochemical mitochondrial gradient by promoting the return of protons to the mitochondrial matrix; this reduces the efficiency of ATP production in the cell [23]. Central nervous system tissues contain higher levels of UCP2 in various types of neurons [24,25,26]. Under physiological conditions, moderate UCP2 activity may protect synaptic integrity and neuronal viability under stress conditions [27,28]. In contrast, during aging or prolonged metabolic stress, sustained elevation of UCP2 expression may impair mitochondrial coupling efficiency, resulting in less efficient energy utilization and reduced metabolic flexibility in neurons [29].

Previous studies have reported that SIRT1 can inhibit UCP2 transcription, thereby contributing to the maintenance of intracellular ATP levels [30]. Increasing evidence suggests that the SIRT1-UCP2 axis participates in the regulation of mitochondrial function and cellular metabolic balance [31]. However, its involvement in the context of natural aging, particularly within the hippocampus, has not been well characterized. Given the close association between age-related declines in mitochondrial efficiency and cognitive impairment, further investigation of the SIRT1-UCP2 regulatory pathway in the aging nervous system may provide insight into the molecular processes contributing to cognitive dysfunction.

Therefore, we examined whether PQQ could mitigate cognitive decline in naturally aged mice by improving hippocampal mitochondrial function and energy metabolic balance. Based on transcriptomic prediction and previous evidence linking SIRT1 and UCP2 to mitochondrial bioenergetics, we further explored whether SIRT1-associated UCP2 regulation may participate in the effects of PQQ.

## 2. Materials and Methods

### 2.1. Animal Experiments

The animal experimental procedures involved in this study were approved by the Ethics Committee for Animal Experimentation of Beijing University of Chinese Medicine (Approval No. BUCM-2024032503-1181). Twenty-month-old male C57BL/6J mice were purchased from Hangzhou Ziyuan Laboratory Animal Technology Co., Ltd. (license number: SCXK [Zhe] 2024-0004; Hangzhou, China), and housed in the SPF animal facility of Beijing University of Chinese Medicine under stable environmental conditions: temperature, 20–24 °C; humidity, 40–70%; and a 12 h light/dark cycle. Mice were provided ad libitum access to standard laboratory chow and water. No caloric restriction or special dietary intervention was applied during the study. Because the mice were purchased as naturally aged animals, detailed lifetime food-intake records before the start of the experiment were not available.

A total of eighteen mice were randomly assigned to three groups (*n* = 6 per group): control (0.2 mL normal saline, CON), low-dose PQQ (5 mg/kg/day in 0.2 mL normal saline, PQQ-L), and high-dose PQQ (10 mg/kg/day in 0.2 mL normal saline, PQQ-H). The doses of PQQ (Cat. No. 00420230403; Zhucheng Haotian Pharm Co., Ltd., Zhucheng, China) used in this study were selected based on previous studies reporting the biological effects of PQQ in mice, together with feasibility considerations for short-term oral gavage in naturally aged mice. The intervention lasted 14 days with oral gavage. After randomization, no obvious differences in food intake or general health status were observed among the CON, PQQ-L, and PQQ-H groups during the intervention period.

### 2.2. Behavioral Assessments

#### 2.2.1. Y-Maze Test

The Y-maze has three arms arranged at 120°, each arm being 300 mm in length, 60 mm in width, and 200 mm in height (YAN-YM; Beijing Zhongshi Dichuang Technology, Beijing, China). On the day before testing, mice were acclimated to the behavior suite to minimize environmental stress. During the test, each mouse was placed in the maze and allowed to explore for 10 min. Locomotor activity was captured by an overhead camera and evaluated with automated tracking software. Entry into three consecutive different arms was defined as spontaneous alternation. The spontaneous alternation percentage was calculated as follows: [Number of alternations/(Total arm entries − 2)] × 100.

#### 2.2.2. Open-Field Test (OFT)

The OFT was employed to evaluate locomotor activity and anxiety-related behavior in mice. Twenty-four hours after the last administration, mice were allowed to explore in an arena (50 cm × 50 cm × 35 cm; length × width × height) for 10 min while their activity was recorded and analyzed by video-tracking software. Total distance traveled and time spent in the center were used as the primary behavioral parameters.

#### 2.2.3. Novel Object Recognition (Nor)

The NOR test was conducted in a square open-field apparatus (40 cm × 40 cm × 40 cm; length × width × height). On day 1, the mice were individually placed in the open field to habituate to the environment for 10 min. On day 2, two identical objects were positioned in diagonally opposite corners of the arena, and mice were allowed to freely explore them for 10 min. Following a 1 h interval, one familiar object was substituted with a novel counterpart, and mice were then permitted to explore for an additional 10 min. Exploration was registered when the mouse’s nose was oriented within 3 cm of an object. Video-tracking software was employed to monitor activity, and the discrimination index was calculated to assess recognition memory.

### 2.3. Histological Analysis

Following completion of the behavioral assessments, mice were deprived of food for 12 h while maintaining free access to water. Animals were subsequently euthanized and perfused transcardially with physiological saline, followed by fixation using 4% paraformaldehyde. Brains were promptly harvested, and the hippocampus and cerebral cortex were isolated on ice, and either snap-frozen or post-fixed according to subsequent experimental requirements.

Brain tissues underwent fixation in 4% paraformaldehyde and subsequent paraffin embedding. Following 4-μm microtome sectioning, hematoxylin and eosin (H&E) staining was employed to evaluate hippocampal cytoarchitecture organization, and Nissl staining was conducted to evaluate neuronal integrity.

### 2.4. JC-1 and ROS Assays

Mouse hippocampal neuronal HT-22 cells were obtained from Wuhan Procell Life Science & Technology Co., Ltd. (Cat. No. CL-0595; Wuhan, China), and cultured under standard conditions. Cells were allocated into three experimental groups: control, H_2_O_2_ model, and H_2_O_2_ + PQQ treatment groups. To establish the oxidative stress model, cells were exposed to H_2_O_2_ at a final concentration of 700 μM for 6 h. For PQQ-treated groups, the H_2_O_2_-containing medium was removed after exposure, and cells were subsequently incubated with fresh medium supplemented with PQQ (12.5 μM or 25 μM) for 12 h.

To evaluate the mitochondrial membrane potential (ΔΨm), cells were stained with the JC-1 fluorescent probe (HY-K0601; MedChemExpress, Monmouth Junction, NJ, USA) according to the provided guidelines. Fluorescence levels were detected utilizing a microplate reader, and the ΔΨm was calculated as the ratio of red to green fluorescence intensity.

Intracellular ROS levels were assessed using the DCFH-DA fluorescent probe (HY-D0940; MedChemExpress, Monmouth Junction, NJ, USA). Following DCFH-DA incubation, cells were washed to remove excess probe, and fluorescence was subsequently measured using a microplate reader. Relative ROS levels were normalized to those of the control group. These fluorescence-based assays were performed and interpreted according to established methodological principles for assessing mitochondrial membrane potential and intracellular ROS levels [32].

### 2.5. Seahorse XF Mitochondrial Respiration

HT-22 cells were plated into Seahorse XF96 culture microplates at a density of 5 × 10^3^ cells. Following cell attachment, the cells were then exposed to PQQ at 12.5, 25, and 50 μM. Before the determination, the culture medium was exchanged for Seahorse XF base medium. The plates were subsequently equilibrated in a CO_2_-free incubator at 37 °C for 1 h before analysis.

Mitochondrial respiratory function was determined via the Cell Mitochondrial Stress Test Kit (Cat. No. 103015-100; Agilent Technologies, Santa Clara, CA, USA), according to the provided guidelines. The Oxygen Consumption Rate (OCR) was monitored while 1 μM oligomycin, 0.5 μM FCCP, and 0.5 μM rotenone/antimycin A were sequentially injected to assess key respiratory parameters.

OCR values were normalized to cell area quantified by the IncuCyte S3 live-cell imaging system. Normalized data were imported into Seahorse Wave software for data correction, and mitochondrial respiration parameters were automatically calculated, exported, and used for downstream analysis and visualization. The Seahorse XF assay was performed and interpreted according to established methodological principles for extracellular flux analysis and mitochondrial respiration assessment [33].

### 2.6. Analysis of RNA-Seq Data

Transcriptome sequencing was executed on the Illumina NovaSeq 6000 platform. Following raw data filtration with fastp, the processed reads were subsequently mapped to the mouse reference genome with HISAT2. Transcript abundance was generated using HTSeq-count, and differential expression was determined through the DESeq2 package. Genes meeting the thresholds of |log2FC| > 1 and q-value < 0.05 were identified as differentially expressed genes (DEGs). Gene Ontology (GO) enrichment analysis was performed for upregulated DEGs using R. GO terms from the biological process (BP), cellular component (CC), and molecular function (MF) categories were analyzed. For visualization in the main figure, the top enriched GO terms ranked by *p*-value were displayed. The complete GO enrichment results for DEGs are provided in Supplementary Figure S1.

### 2.7. Ingenuity Pathway Analysis (IPA)

IPA was used to identify upstream regulators, biological functions, and canonical pathways associated with the DEGs. DEGs meeting the thresholds of |log2FC| > 1 and q-value < 0.05 were uploaded into IPA, and core analyses were performed using the default settings. Activation z-scores and *p*-values derived from the IPA database were used to predict the activation or inhibition of pathways. The results were visualized through bar plots, network diagrams, and heatmaps generated in the IPA platform.

### 2.8. Molecular Docking

The three-dimensional coordinates of mouse SIRT1 were retrieved from the Protein Data Bank, while the chemical structure of PQQ was retrieved from PubChem. Before docking, the protein was preprocessed by eliminating water molecules, incorporating hydrogen atoms, and calculating Gasteiger charges.

Docking simulations were carried out using AutoDock Vina (version 1.1.2). The docking grid was positioned to encompass the predicted ligand-binding pocket of SIRT1, and simulations were conducted with default settings. The docking exhibiting the minimum binding energy was selected as the optimal conformation. Protein–ligand interactions were displayed and evaluated using PyMOL (version 2.3.0) and Discovery Studio Visualizer (version 21.1.20298).

### 2.9. ATP Measurement

HT-22 cells were cultured under standard conditions and seeded into 12-well plates at a density of 8 × 10^4^ cells per well. After 24 h of attachment, cells were treated according to experimental grouping. EX-527 (300 μM; Cat. No. HY-15452; MedChemExpress, Monmouth Junction, NJ, USA) was applied for 9 h, followed by PQQ treatment (12.5 μM) for 12 h where indicated. Intracellular ATP levels were measured using a commercial ATP assay kit (HY-K0314; MedChemExpress, Monmouth Junction, NJ, USA) according to the manufacturer’s instructions.

### 2.10. RT-qPCR and Western Blot

Following TRIzol-based RNA extraction, cDNA was synthesized for subsequent quantitative PCR analysis using a reverse transcription kit. Relative mRNA expression was normalized to ACTIN and quantified using the 2-ΔΔCt method. Primer sequences: ACTIN (Up: TTGTGTAAGGTAAGGTGTGC, Down: GGTTGAGGTGTTGAGGCAG), UCP2 (Up: ATGGTTGGTTTCAAGGCCACA, Down: CGGTATCCAGAGGGAAAGTGAT), CD74 (Up: CCGCCTAGACAAGCTGACC, Down: CAGGTTTGGCAGATTTCGGA), SFRP5 (Up: CACTGCCACAAGTTCCCCC, Down: TCTGTTCCATGAGGCCATCAG).

For Western blot analysis, hippocampal proteins were extracted and quantified using the BCA method. Equal amounts of the proteins were subjected to SDS-PAGE and transferred to a PVDF membrane. After blocking, membranes were incubated with primary antibodies against SIRT1 (1:1000; CST), UCP2 (1:1000; Selleck), and GAPDH (1:10,000; Selleck), and subsequent HRP-conjugated secondary antibodies. Protein bands were detected by ECL and quantified using ImageJ (version 1.50).

### 2.11. Statistical Analysis

Statistical analysis was conducted with GraphPad Prism 9, and data were expressed as mean ± SEM. Comparisons between two groups were conducted using unpaired two-tailed Student’s *t*-tests, while multiple group comparisons were executed using one-way ANOVA followed by Tukey’s post hoc test. Statistical significance was defined as * *p* < 0.05, ** *p* < 0.01, *** *p* < 0.001.

## 3. Results

### 3.1. PQQ Improves Cognitive-Related Behavioral Performance in Naturally Aged Mice

**Figure 1:**
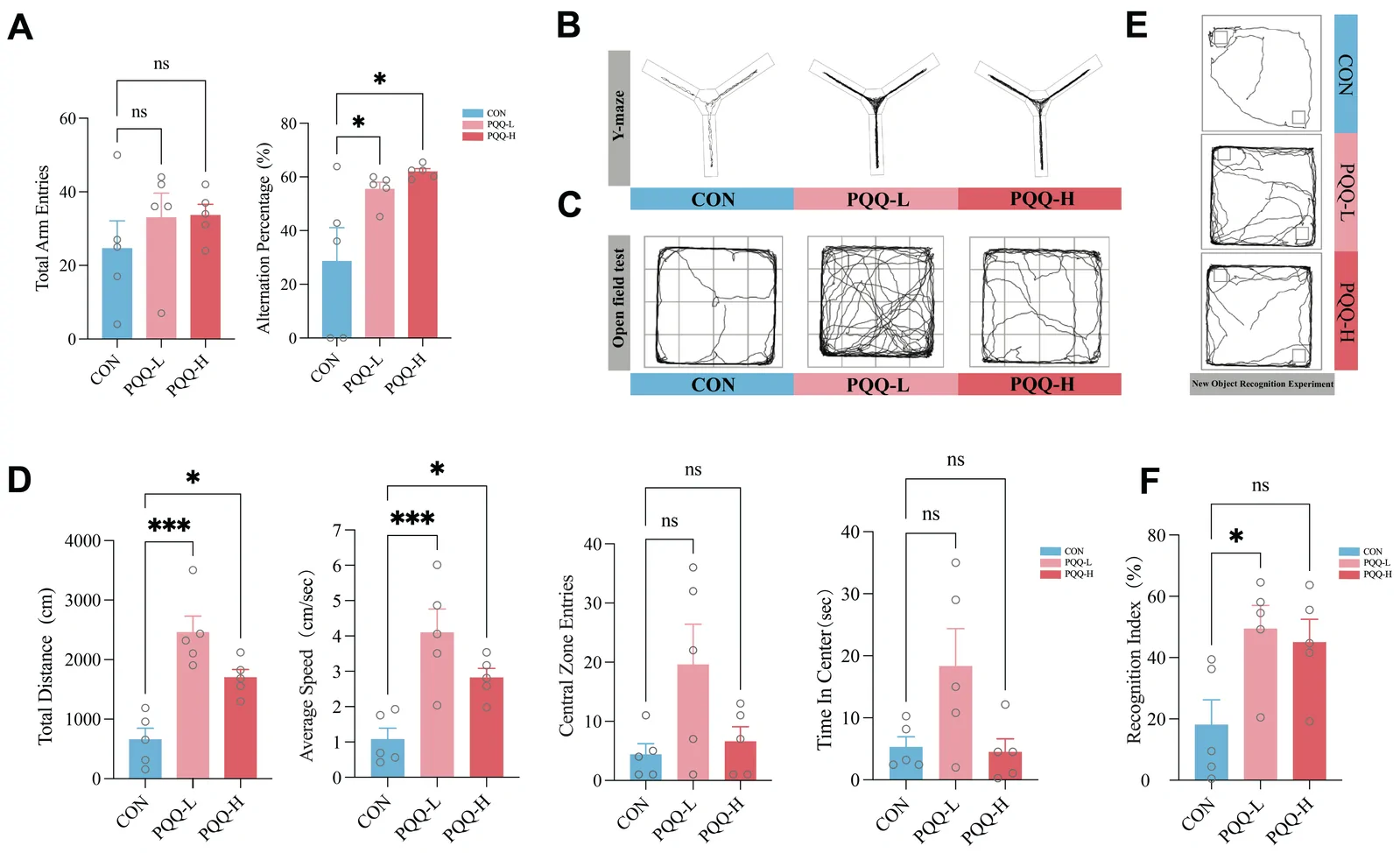
PQQ improves spatial and recognition memory in naturally aged mice. (**A**) Y-maze performance parameters (*n* = 5). (**B**) Representative Y-maze exploration trajectories (*n* = 5). (**C**) Representative trajectories from the OFT (*n* = 5). (**D**) Quantification of open field parameters, including total distance traveled, average speed, central zone entries, and time spent in the center (*n* = 5). (**E**) Representative exploration trajectories in the NOR test (*n* = 5). (**F**) Recognition index in the NOR test (*n* = 5). Data are presented as the mean ± SEM. ns, not significant. * *p* < 0.05; *** *p* < 0.001.

Spatial working memory was assessed using the spontaneous alternation test in the Y-maze. As shown in Figure 1A, both low-dose and high-dose PQQ treatments significantly increased the spontaneous alternation percentage in naturally aged mice relative to controls, without affecting total arm entries, suggesting an improvement in spatial working memory rather than a mere change in locomotor activity. Representative exploration trajectories further revealed more organized alternation patterns in PQQ-treated mice (Figure 1B).

In the open-field test, open-field trajectories are shown in Figure 1C. Both low-dose and high-dose PQQ treatments significantly increased total distance traveled and average movement speed in aged mice compared with the control group (Figure 1D). In contrast, no significant differences were observed among groups in either central zone entries or time spent in the center, suggesting that PQQ treatment enhanced locomotor activity without affecting anxiety-related behavior (Figure 1D).

Recognition memory was evaluated using the NOR test. As illustrated in Figure 1F, mice in the PQQ-L group exhibited a higher recognition index compared with control mice, whereas mice in the PQQ-H group showed an increasing trend that did not reach statistical significance. Representative exploration trajectories for each group are presented in Figure 1E.

Collectively, these results suggest that PQQ improves performance in hippocampus-related behavioral tasks in naturally aged mice.

### 3.2. PQQ Preserves Hippocampal Neuronal Integrity

**Figure 2:**
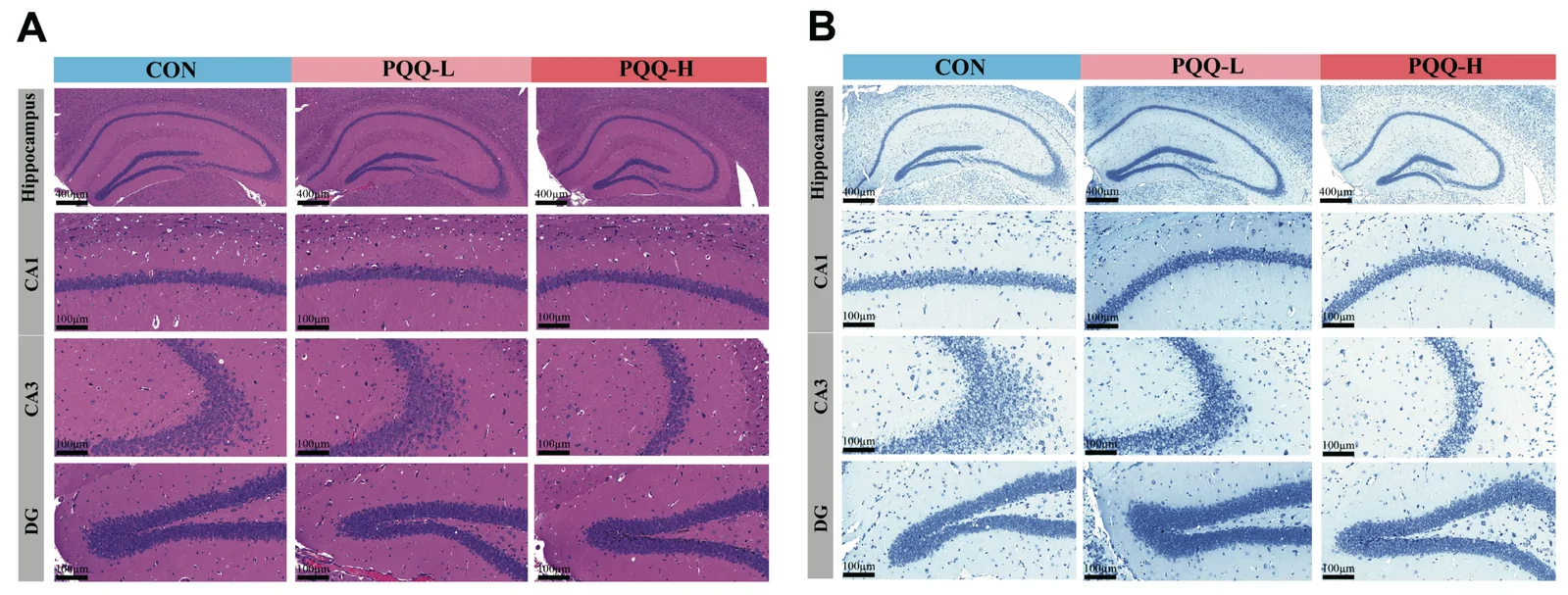
PQQ preserves hippocampal histological structure in naturally aged mice. (**A**) Representative H&E staining of hippocampal sections, including CA1, CA3, and DG regions. (**B**) Representative Nissl staining of hippocampal subregions. Scale bars: 400 μm (hippocampus) and 100 μm (CA1, CA3, DG).

Hematoxylin and eosin (H&E) staining was used to evaluate hippocampal morphology. Representative images from control mice showed apparent age-related neuropathological alterations, including neuronal soma shrinkage and disrupted cellular organization across the Cornu Ammonis 1 (CA1), Cornu Ammonis 3 (CA3), and dentate gyrus (DG) regions (Figure 2A). In contrast, representative images from the PQQ-L and PQQ-H groups showed relatively preserved hippocampal morphology, characterized by more organized pyramidal and granule cell layers and fewer apparent morphological features suggestive of neuronal degeneration.

Consistent with the H&E observations, Nissl staining showed reduced Nissl substance intensity and a loosely organized neuronal arrangement across hippocampal subregions in representative images from control mice (Figure 2B), suggesting compromised neuronal integrity. In the PQQ-treated groups, representative images showed clearer neuronal boundaries, relatively stronger Nissl staining, and a more compact arrangement of neurons in the CA1, CA3, and DG regions.

Together, the representative histological observations suggest that PQQ treatment may be associated with better preservation of hippocampal neuronal morphology in naturally aged mice.

### 3.3. PQQ Induces Hippocampal Transcriptomic Remodeling

**Figure 3:**
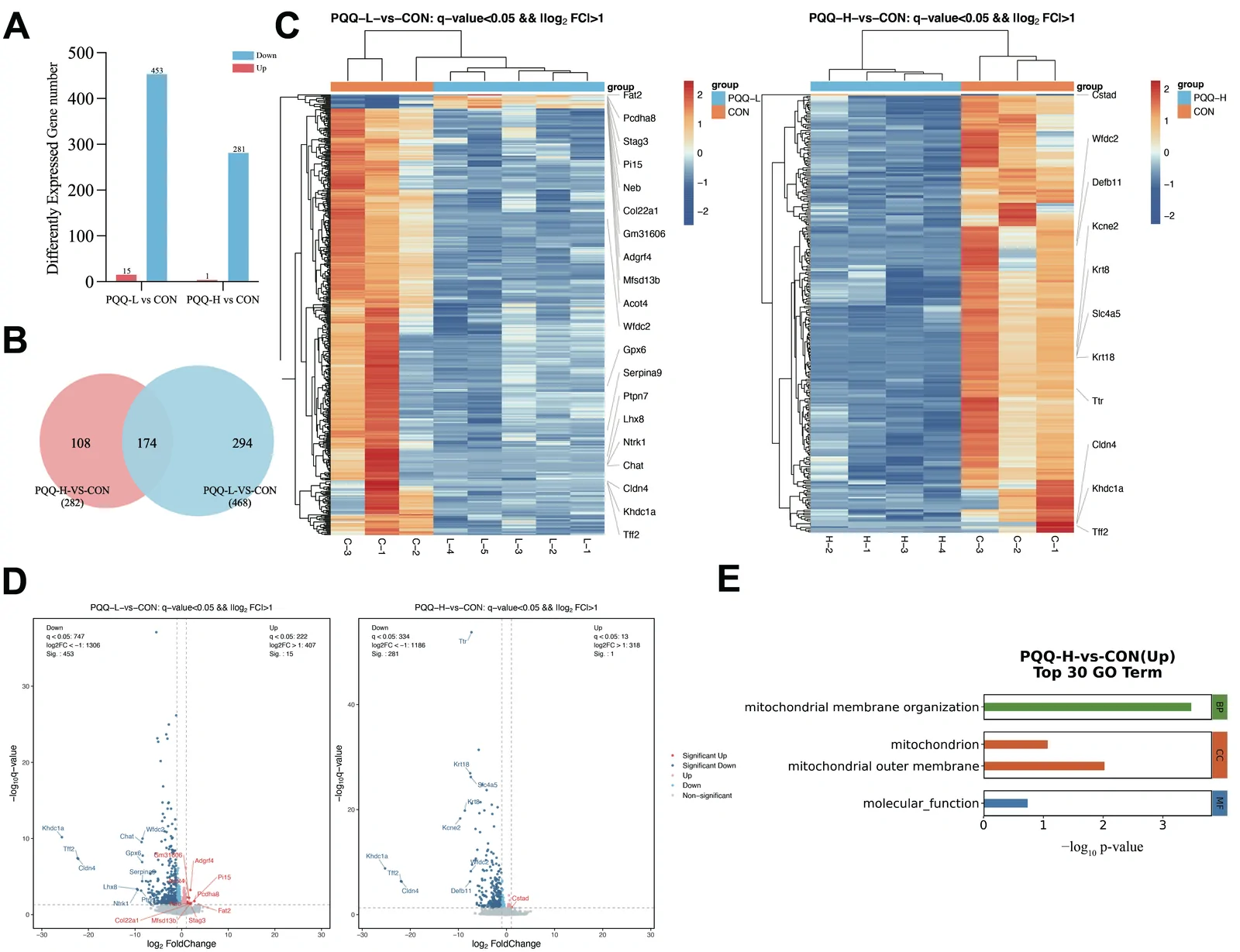
PQQ modulates the hippocampal transcriptome in naturally aged mice. (**A**) DEGs identified in the PQQ-L and PQQ-H groups compared with the control group. (**B**) Venn diagram showing the overlap of DEGs between the PQQ-L vs. control and PQQ-H vs. control comparisons. (**C**) Heatmaps showing hierarchical clustering of DEGs across groups. (**D**) Volcano plots displaying DEGs in PQQ-treated groups relative to controls. (**E**) GO enrichment analysis of upregulated DEGs.

RNA sequencing (RNA-seq) was performed to characterize hippocampal transcriptomic alterations following PQQ treatment in aged mice. As shown in Figure 3A, analysis of differentially expressed genes (DEGs) revealed 468 DEGs in the PQQ-L group and 282 DEGs in the PQQ-H group compared with controls, with the majority of DEGs being downregulated in both comparison groups. Through Venn analysis, we identified 174 overlapping DEGs between the two treatment groups, indicating a common core transcriptional response (Figure 3B).

Hierarchical clustering of the DEGs revealed a distinct segregation between PQQ-treated samples (both low and high doses) and the control group, with high within-group consistency, indicating the robustness and reproducibility of the transcriptional remodeling elicited by PQQ (Figure 3C). Volcano plot analysis showed a predominance of downregulated transcripts in both the PQQ-L and PQQ-H groups, and these downregulated genes were primarily associated with neurons, cell structure, and membrane transport (Figure 3D). Gene Ontology (GO) enrichment analysis revealed that upregulated genes were mainly enriched in biological processes related to mitochondrial structure and membrane integrity (Figure 3E).

### 3.4. PQQ Protects Mitochondrial Function Against Oxidative Injury in HT-22 Cells

**Figure 4:**
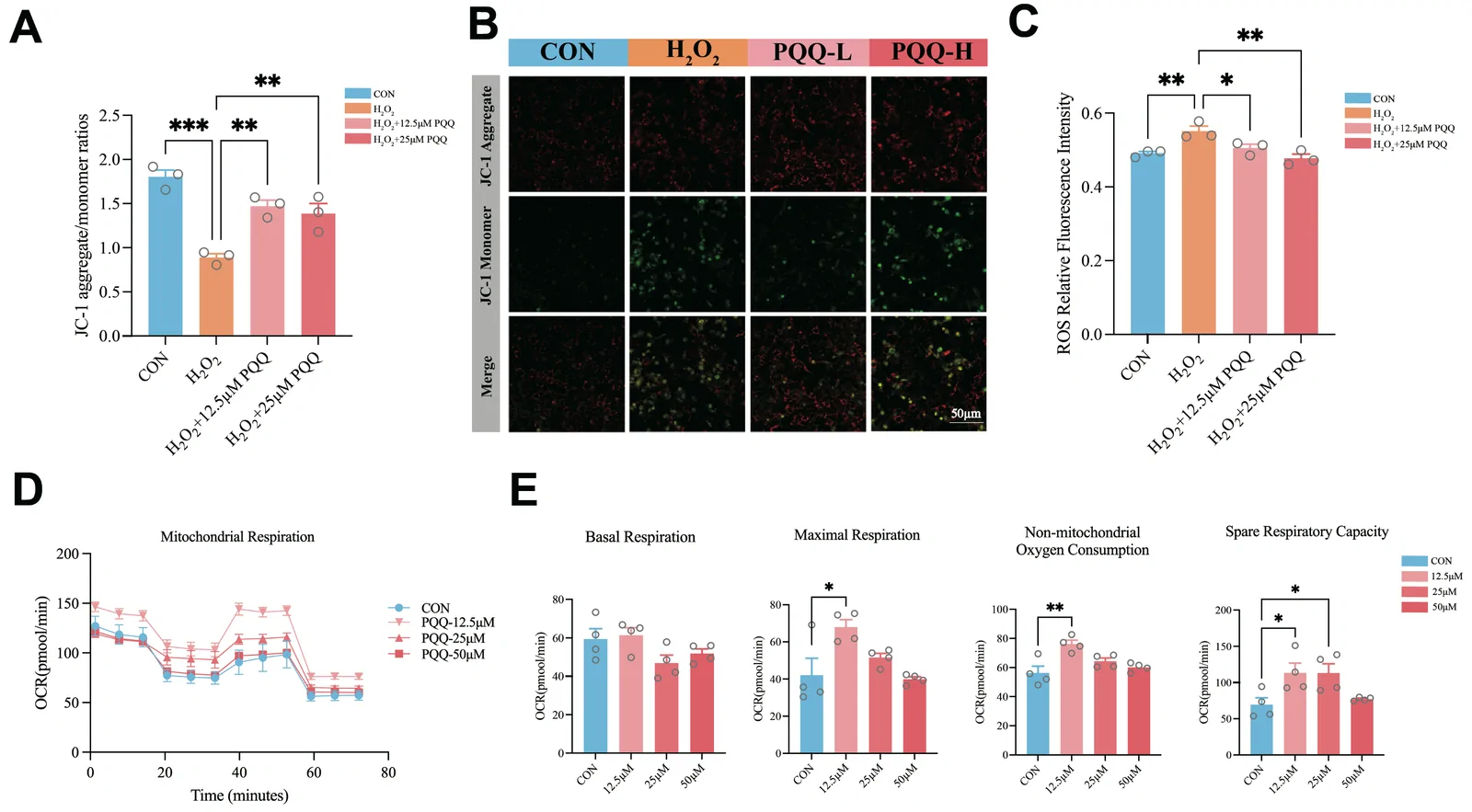
PQQ improves mitochondrial function in HT-22 neuronal cells under oxidative stress. (**A**) Quantification of mitochondrial membrane potential assessed by JC-1 aggregate-to-monomer ratio in HT-22 cells following oxidative stress (*n* = 3). (**B**) Representative JC-1 fluorescence images. (**C**) Quantification of intracellular ROS levels in HT-22 cells (*n* = 3). (**D**) OCR profiles measured using the Seahorse XF Cell Mito Stress Test (*n* = 4). (**E**) Quantification of mitochondrial respiration parameters (*n* = 4). Data are presented as the mean ± SEM. * *p* < 0.05; ** *p* < 0.01; *** *p* < 0.001.

To validate the transcriptomic results, we further evaluated mitochondrial function in vitro using HT-22 cells. JC-1 staining results showed that the H_2_O_2_ exposure significantly diminished the ratio of JC-1 aggregates to monomers, indicating that the mitochondrial membrane potential was damaged. However, this ratio was significantly restored after PQQ treatment (Figure 4A,B), suggesting that PQQ can effectively alleviate the loss of mitochondrial membrane potential. In addition, PQQ also exhibited antioxidant ability, which can significantly clear the accumulation of intracellular ROS induced by H_2_O_2_ (Figure 4C).

### 3.5. PQQ Enhances Mitochondrial Respiratory Capacity in HT-22 Cells

Mitochondrial respiratory function in HT-22 cells was analyzed using the Seahorse XF assay. As shown in Figure 4D, treatment with 12.5 μM PQQ elevated the oxygen consumption rate (OCR) throughout the entire respiratory profile. While 25 μM PQQ also increased OCR relative to control, the stimulatory effect was less pronounced than that observed at the 12.5 μM dose. In contrast, 50 μM PQQ produced minimal effects and failed to significantly enhance OCR compared with controls.

Quantitative analysis further confirmed that lower concentrations of PQQ significantly increased maximal respiration, non-mitochondrial oxygen consumption, and spare respiratory capacity (Figure 4E). Basal respiration remained comparable among all experimental groups. Collectively, these results indicate that PQQ improves mitochondrial respiratory performance in HT-22 cells, with the most pronounced effects observed at lower concentrations.

### 3.6. PQQ Is Associated with Activation of SIRT1-Related Regulatory Signaling and Suppression of UCP2

**Figure 5:**
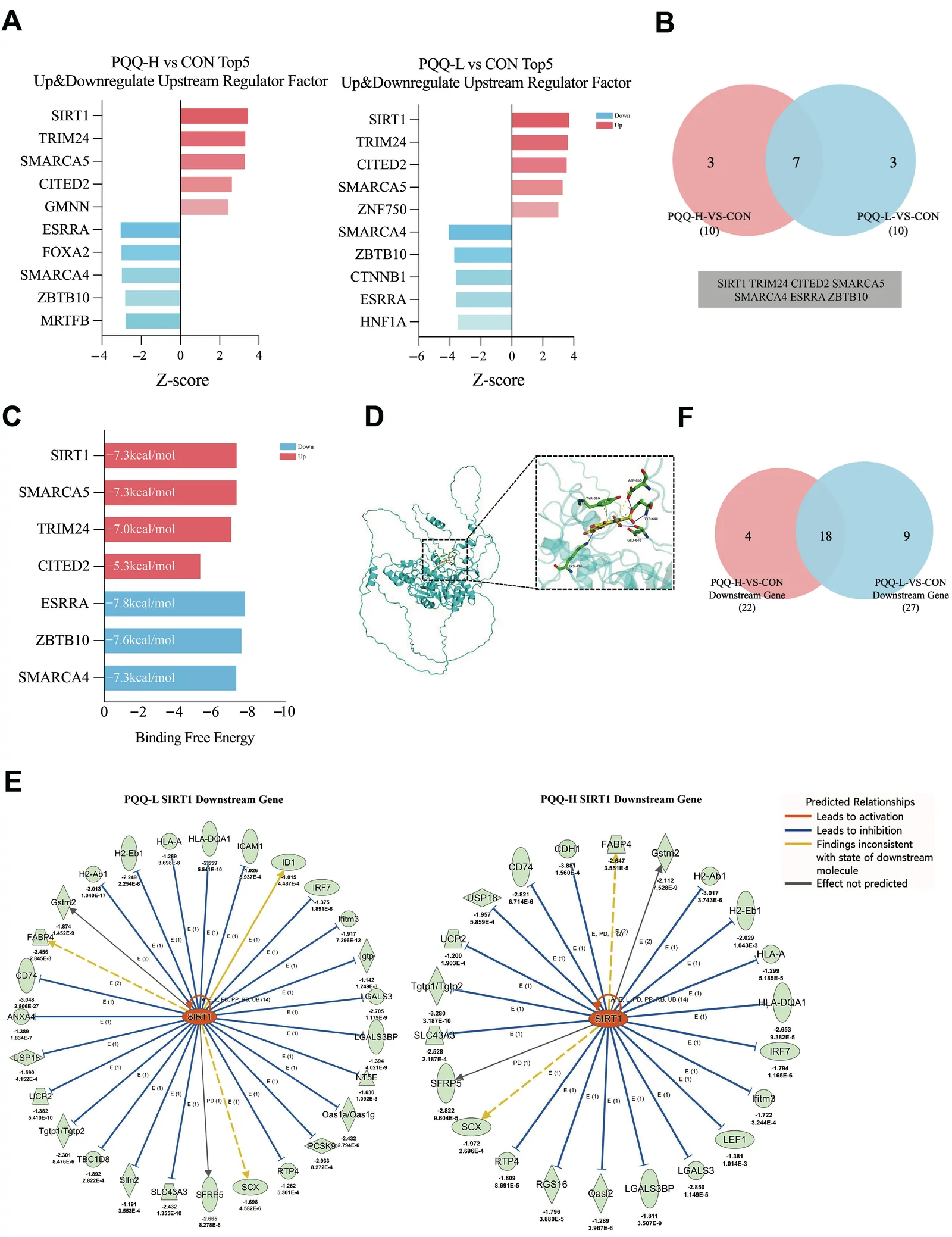
PQQ treatment is associated with SIRT1-related regulatory signaling and selected downstream transcriptomic changes. (**A**) Top predicted upstream regulators identified by IPA in PQQ-treated groups based on activation z-scores. (**B**) Overlap of predicted upstream regulators between PQQ-L and PQQ-H groups. (**C**) Predicted binding free energies of PQQ with candidate upstream regulators. (**D**) Representative molecular docking model illustrating the interaction between PQQ and SIRT1. (**E**) IPA-derived SIRT1 downstream gene networks in PQQ-treated groups. (**F**) Overlap of SIRT1 downstream genes between PQQ-L and PQQ-H groups.

To identify potential upstream regulators involved in the hippocampal transcriptional response to PQQ, IPA-based upstream regulator analysis was performed using the identified DEGs. In the comparison between the PQQ-H and Control (CON) groups, SIRT1 showed the highest predicted activation z-score among the top upstream regulators, followed by TRIM24, SMARCA5, CITED2, and GMNN, suggesting that SIRT1-related regulatory signaling may be involved in the molecular response to PQQ treatment. In contrast, several regulators, including ESRRA, FOXA2, SMARCA4, ZBTB10, and MRTFB, showed negative z-scores, indicating predicted inhibition (Figure 5A). A similar regulatory pattern was observed in the PQQ-L group, in which SIRT1 was also predicted as one of the most prominently activated upstream regulators. Based on these results, SIRT1 was prioritized for further validation for several reasons. First, IPA upstream regulator analysis identified SIRT1 as a predicted upstream regulator associated with the PQQ-induced transcriptional response. Second, SIRT1 is closely related to mitochondrial homeostasis, redox regulation, energy metabolism, and aging-associated adaptation.

Venn diagram analysis identified seven common predicted upstream regulators between the PQQ-L vs. CON and PQQ-H vs. CON comparisons, including SIRT1, TRIM24, CITED2, SMARCA5, SMARCA4, ESRRA, and ZBTB10 (Figure 5B). To further explore the potential interaction between PQQ and these candidate regulators, molecular docking analysis was performed. Among the candidate regulators, SIRT1 exhibited favorable predicted binding affinity with PQQ, suggesting a possible structural basis for the association between PQQ treatment and SIRT1-related signaling (Figure 5C,D).

Based on these findings, we further examined the SIRT1-associated downstream regulatory network predicted by IPA. IPA identified 18 overlapping SIRT1-associated downstream genes that were consistently suppressed in both PQQ-treated groups (Figure 5E), including CD74, FABP4, Gstm2, H2-Ab1, H2-Eb1, HLA-A, HLA-DQA1, Ifitm3, IRF7, LGALS3, LGALS3BP, RTP4, SCX, SFRP5, SLC43A3, Tgtp1/Tgtp2, UCP2, and USP18 (Figure 5F). Although several inflammation-, neuroimmune-, and metabolism-related genes showed prominent changes, UCP2 was selected for further validation because it is a SIRT1-associated downstream candidate directly involved in mitochondrial proton conductance and bioenergetic efficiency. This biological function matched the mitochondrial functional assays performed in this study. Therefore, the SIRT1–UCP2-associated pathway was selected as a focused candidate mechanism for validation.

**Figure 6:**
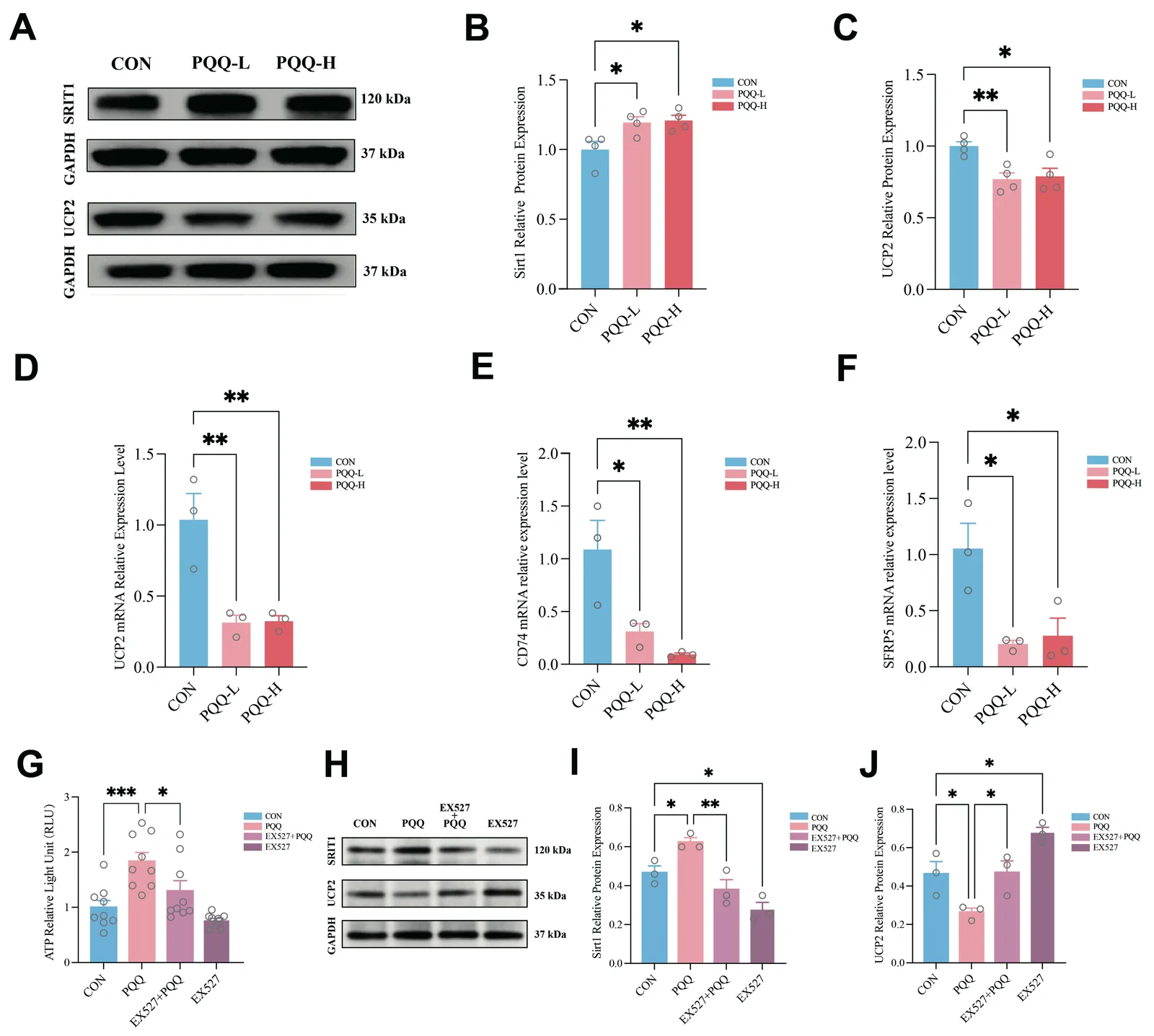
Experimental validation of SIRT1/UCP2-associated regulation after PQQ treatment. (**A**) Representative Western blot images of SIRT1 and UCP2 in hippocampal tissue. (**B**) Quantification of relative SIRT1 protein expression (*n* = 4). (**C**) Quantification of relative UCP2 protein expression (*n* = 4). (**D**) Relative UCP2 mRNA expression measured by RT-qPCR (*n* = 3). (**E**) Relative CD74 mRNA expression measured by RT-qPCR (*n* = 3). (**F**) Relative SFRP5 mRNA expression measured by RT-qPCR (*n* = 3). (**G**) Intracellular ATP levels in HT-22 cells treated with PQQ in the presence or absence of the SIRT1 inhibitor EX-527 (*n* = 9). (**H**) Representative Western blot images of SIRT1 and UCP2. (**I**) Quantification of relative SIRT1 protein expression (*n* = 3). (**J**) Quantification of relative UCP2 protein expression (*n* = 3). Data are presented as the mean ± SEM. * *p* < 0.05; ** *p* < 0.01; *** *p* < 0.001.

We next examined SIRT1 and UCP2 expression in hippocampal tissue. Western blot analysis showed that both PQQ-L and PQQ-H treatments significantly increased hippocampal SIRT1 protein expression compared with the CON group, whereas UCP2 protein expression was significantly reduced (Figure 6A–C). Consistent with the protein-level changes, RT-qPCR analysis showed that UCP2 mRNA expression was also significantly decreased in both PQQ-treated groups (Figure 6D). These results indicate that PQQ treatment is associated with increased SIRT1 expression and reduced UCP2 expression in the hippocampus of naturally aged mice.

To further validate additional RNA-seq-derived candidates from the SIRT1-associated downstream network, SFRP5 and CD74 were examined by RT-qPCR. These genes were selected based on their differential expression and biological relevance to neuroimmune responses, metabolic regulation, and SIRT1-associated signaling. The RT-qPCR results showed that both SFRP5 and CD74 were significantly decreased in the PQQ-treated groups, which was generally consistent with the RNA-seq trends (Figure 6E,F). These findings provide additional validation for selected transcriptomic changes induced by PQQ treatment.

To further evaluate whether SIRT1 participates in PQQ-associated mitochondrial bioenergetic regulation, the selective SIRT1 inhibitor EX-527 was applied in HT-22 cells. PQQ treatment significantly increased intracellular ATP levels compared with the control group, whereas co-treatment with EX-527 markedly attenuated this increase (Figure 6G). These findings suggest that SIRT1 activity contributes to the PQQ-associated enhancement of cellular bioenergetic output.

Western blot analysis further showed that PQQ increased SIRT1 protein expression and reduced UCP2 protein expression in HT-22 cells. However, the regulatory effects of PQQ on both SIRT1 and UCP2 were weakened in the presence of EX-527 (Figure 6H–J). Together, these results support the involvement of SIRT1 in PQQ-associated mitochondrial regulation and suggest that suppression of UCP2 may represent a downstream component of this regulatory response.

## 4. Discussion

Age-related cognitive decline is closely associated with impaired hippocampal function, neuronal structural deterioration, oxidative stress, and mitochondrial dysfunction [34,35]. Because hippocampal neurons have high energy demands, disruption of mitochondrial bioenergetics may contribute substantially to the progressive decline in learning and memory during aging [36,37]. In the present study, we used a naturally aged mouse model to evaluate the potential neuroprotective effects of PQQ. Our results showed that PQQ improved performance in hippocampus-related behavioral tasks, preserved hippocampal neuronal morphology, enhanced mitochondrial function in HT-22 neuronal cells, and was associated with modulation of SIRT1- and UCP2-related mitochondrial regulatory signaling.

Unlike acute aging models induced by D-galactose or other chemical stressors, natural aging models more closely reflect the gradual physiological deterioration that occurs over time. In this study, twenty-month-old naturally aged mice were used to examine whether PQQ could improve cognitive performance under aging-related conditions. PQQ supplementation increased spontaneous alternation in the Y-maze and improved recognition performance in the novel object recognition task, suggesting beneficial effects on spatial working memory and recognition memory. Importantly, PQQ did not significantly alter center time or center entries in the open-field test, indicating that the observed behavioral changes were unlikely to be driven by increased anxiety-like behavior. However, PQQ increased total distance traveled and average movement speed, indicating enhanced locomotor activity. Although spontaneous alternation and recognition index are relative measures that are less dependent on absolute movement distance, locomotor changes may still influence behavioral performance. Therefore, the behavioral findings should be interpreted as improved cognitive-related task performance rather than definitive reversal of age-related cognitive decline. Future studies using additional behavioral paradigms less sensitive to locomotor activity will help further clarify the cognitive effects of PQQ. H&E and Nissl staining showed that PQQ-treated mice exhibited better preservation of hippocampal cytoarchitecture, clearer neuronal boundaries, and increased Nissl compared with aged control mice.

To explore the molecular basis underlying the effects of PQQ, we performed hippocampal transcriptomic analysis. RNA-seq revealed that both low- and high-dose PQQ induced clear transcriptional remodeling in the hippocampus, with overlapping differentially expressed genes between the two treatment groups. Interestingly, the transcriptional response was dominated by downregulated genes, whereas the relatively fewer upregulated genes were enriched in processes related to mitochondrial structure, membrane organization, and energy metabolism. This pattern suggests that PQQ may not simply activate a broad transcriptional program, but may instead reshape aging-associated transcriptional disturbances while selectively supporting mitochondrial-related biological processes. Because aging is often accompanied by increased transcriptional noise and dysregulated stress-response signaling [38], the predominance of downregulated genes may reflect partial normalization of maladaptive or compensatory transcriptional changes in the aging hippocampus. However, this interpretation remains hypothesis-generating and requires further validation using independent datasets and targeted molecular assays.

The mitochondrial relevance of the transcriptomic findings was supported by in vitro functional assays in HT-22 hippocampal neuronal cells. Under H_2_O_2_-induced oxidative stress, PQQ reduced intracellular ROS accumulation and restored mitochondrial membrane potential, indicating improved mitochondrial resilience under oxidative injury. Seahorse XF analysis further showed that PQQ enhanced mitochondrial respiratory capacity, particularly maximal respiration and spare respiratory capacity. These parameters reflect the ability of cells to meet increased energetic demand under stress conditions and are highly relevant to neuronal survival and function. Notably, the respiratory benefit of PQQ was not linearly increased with concentration: 12.5 μM produced the strongest effect, whereas 25 μM showed a weaker effect and 50 μM showed minimal enhancement. This pattern indicates a non-linear concentration-dependent response and is consistent with a possible hormetic-like effect, in which a relatively low dose induces beneficial mitochondrial adaptation whereas higher doses produce weaker or neutral responses. However, additional concentrations, longer exposure durations, and direct assessment of cell viability or stress-response markers are needed to formally establish a complete hormetic dose–response relationship. Therefore, the optimal dose range and long-term safety of PQQ in the aging nervous system should be further investigated.

IPA-based upstream regulator analysis identified SIRT1 as a major predicted upstream regulator associated with the PQQ-induced transcriptional response. Previous studies have linked decreased SIRT1 signaling to aging-associated mitochondrial dysfunction and cognitive impairment [39,40,41,42]. In the present study, PQQ increased hippocampal SIRT1 protein expression, and molecular docking analysis suggested a potential interaction between PQQ and SIRT1. These findings support an association between PQQ treatment and SIRT1-related regulatory signaling. Among the SIRT1-associated downstream genes identified by IPA, UCP2 was selected for further validation because of its established role in mitochondrial uncoupling and energy efficiency. UCP2 can reduce mitochondrial coupling efficiency by facilitating proton conductance across the inner mitochondrial membrane [43]. In this study, PQQ reduced UCP2 expression at both mRNA and protein levels in the hippocampus, which was consistent with improved mitochondrial membrane potential and respiratory capacity observed in HT-22 cells. These findings suggest that suppression of UCP2 may be involved in the PQQ-associated improvement of mitochondrial bioenergetic regulation.

To further examine the involvement of SIRT1, we used the selective SIRT1 inhibitor EX-527 in HT-22 cells. EX-527 attenuated the PQQ-induced increase in ATP production and weakened the regulatory effects of PQQ on SIRT1 and UCP2 protein expression. These results provide functional support for the participation of SIRT1 in PQQ-associated mitochondrial bioenergetic regulation. Nevertheless, these data should not be interpreted as definitive proof that the SIRT1-UCP2 axis is the only or indispensable mechanism underlying the effects of PQQ. PQQ may also influence mitochondrial function through additional pathways, including antioxidant responses, mitochondrial biogenesis, redox regulation, and other metabolic signaling networks.

From a translational perspective, PQQ is a redox-active nutritional compound with reported antioxidant and mitochondrial regulatory properties. The present study extends previous observations by showing that PQQ improves behavioral performance in naturally aged mice and is associated with mitochondrial-related outcomes, thereby providing evidence under a physiological aging context relevant to age-associated cognitive impairment. Nutritional interventions that target mitochondrial and metabolic homeostasis have attracted increasing attention in aging research. Caloric restriction and dietary restriction are among the best-characterized interventions known to improve metabolic flexibility, reduce oxidative stress, and modulate Sirtuin-related signaling pathways [44]. In particular, SIRT1 is considered an important nutrient-sensitive regulator linking energy availability to mitochondrial function, stress resistance, and aging-associated physiological adaptation [45]. PQQ, as a redox-active nutritional compound with mitochondrial regulatory properties, may share conceptual overlap with nutritional strategies aimed at preserving mitochondrial bioenergetics [46]. Previous studies have reported that PQQ can stimulate mitochondrial biogenesis and activate SIRT1/PGC-1α-related signaling [47]. However, unlike caloric restriction, PQQ supplementation did not involve reduced energy intake in the present study. Therefore, the beneficial effects observed here are more likely related to direct or indirect regulation of mitochondrial function and redox homeostasis rather than caloric restriction-like energy limitation.

However, several limitations should be acknowledged. First, no young adult reference group was included, as this study focused on the effects of PQQ within naturally aged mice rather than age-group comparisons. Thus, whether PQQ restores these outcomes toward young adult levels remains unclear. Second, the relatively small sample size in the animal experiments should be noted. Although consistent trends were observed across behavioral, histological, transcriptomic, molecular, and cellular analyses, larger animal cohorts are needed to confirm the reproducibility and generalizability of these findings. Third, the mechanistic evidence for the SIRT1–UCP2-associated pathway remains associative rather than fully causal. Although EX-527 attenuated the PQQ-induced increase in ATP production and weakened the regulatory effects of PQQ on SIRT1 and UCP2 protein expression, UCP2 was not directly manipulated in the present study. Therefore, whether UCP2 is required for the mitochondrial bioenergetic effects of PQQ remains to be established through future knockdown, overexpression, or rescue experiments. Finally, the transcriptomic response to PQQ was not limited to SIRT1–UCP2-associated signaling. Other inflammation-, neuroimmune-, membrane transport-, and metabolic regulation-related genes were also altered. Therefore, the SIRT1–UCP2-associated pathway should be viewed as one candidate regulatory axis selected for focused validation based on its relevance to mitochondrial bioenergetics, rather than as the only mechanism affected by PQQ. Future studies are needed to determine how these additional pathways contribute to the neuroprotective and mitochondrial effects of PQQ.

Despite these limitations, the consistency among behavioral, histological, transcriptomic, molecular, and cellular findings supports a coherent regulatory framework. Collectively, our results indicate that PQQ improves cognitive-related behavioral performance in naturally aged mice and is associated with mitochondrial bioenergetic regulation. These effects are associated with reduced oxidative stress, improved mitochondrial bioenergetic capacity, increased SIRT1 expression, and decreased UCP2 expression. Thus, modulation of a SIRT1-UCP2-associated mitochondrial regulatory pathway may contribute to the neuroprotective effects of PQQ during aging.

## 5. Conclusions

In summary, the present study shows that PQQ supplementation improves cognitive-related behavioral performance in naturally aged mice, particularly in tasks related to spatial working memory and recognition memory. These behavioral effects were accompanied by better preservation of hippocampal neuronal integrity and transcriptomic changes enriched in mitochondrial structure and membrane-related biological processes. In HT-22 hippocampal neuronal cells, PQQ reduced oxidative stress, restored mitochondrial membrane potential, and enhanced mitochondrial respiratory capacity, supporting its role in mitochondrial bioenergetic regulation.

Mechanistically, hippocampal transcriptomic analysis and upstream regulator prediction identified SIRT1-related signaling as a potential regulatory component of the PQQ-associated response. Consistently, PQQ increased SIRT1 protein expression and reduced UCP2 expression in the hippocampus of naturally aged mice. In vitro inhibition of SIRT1 attenuated the PQQ-induced increase in ATP production and partially weakened the regulation of UCP2, suggesting that SIRT1-UCP2-associated signaling may participate in the mitochondrial bioenergetic effects of PQQ.

Taken together, these findings indicate that PQQ improves cognitive-related behavioral performance in naturally aged mice in association with enhanced mitochondrial bioenergetics and reduced oxidative stress. The SIRT1-UCP2-associated pathway may represent a potential regulatory mechanism contributing to these effects, although further studies involving direct manipulation of SIRT1 and UCP2 are required to establish causality. These results provide additional evidence supporting mitochondrial bioenergetic regulation as a promising target for nutritional interventions aimed at mitigating age-associated cognitive functional decline.

## Data Availability

The RNA sequencing data generated in this study have been deposited in the Gene Expression Omnibus (GEO) database under accession number GSE338219. Other data supporting the findings of this study are included in the article and Supplementary Materials.

## Supplementary Materials

The following supporting information can be downloaded at: https://www.mdpi.com/article/10.3390/antiox15080921/s1, Figure S1: GO enrichment analysis of downregulated DEGs identified in PQQ-treated groups. (A) PQQ-H vs. CON. (B) PQQ-L vs. CON.
