# Supplementary material for: Pyrroloquinoline Quinone Improves Cognitive-Related Behavioral Performance Associated with Enhanced Mitochondrial Bioenergetics in Naturally Aged Mice
Source: Antioxidants (Basel). 2026 Jul 24;15(8):921. doi: 10.3390/antiox15080921 (PMC13509254; doi:10.3390/antiox15080921)

Figure S1: GO enrichment analysis of downregulated DEGs identified in PQQ-treated groups. (A) PQQ-H vs. CON. (B) PQQ-L vs. CON.

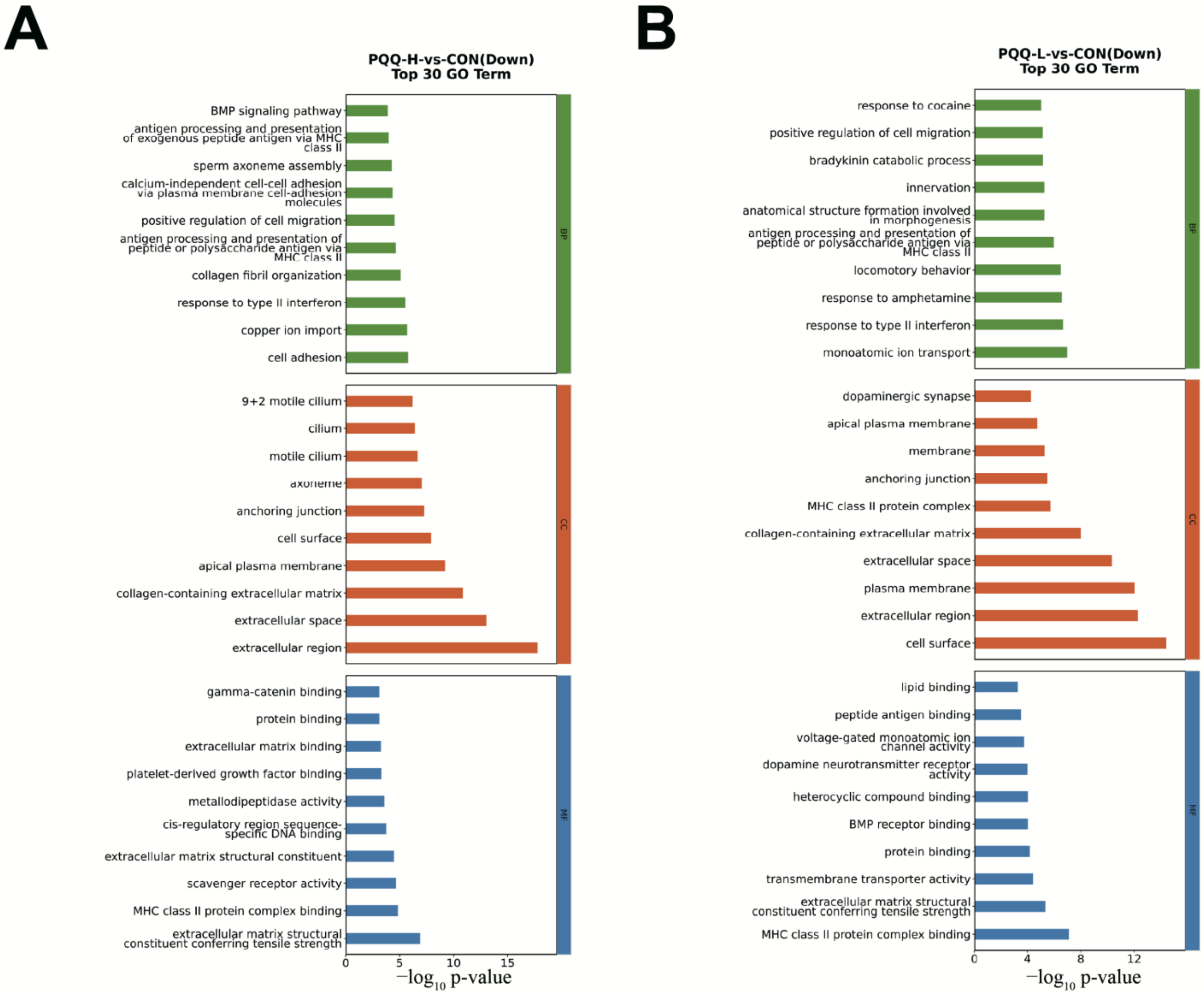

Supplement: Supplementary file 1 [file antioxidants-15-00921-s001.zip › antioxidants-4359133-supplementary.pdf]
